# Natural Bioactive Compounds in the Management of Periodontal Diseases: A Comprehensive Review

**DOI:** 10.3390/molecules29133044

**Published:** 2024-06-26

**Authors:** Nada Tawfig Hashim, Rasha Babiker, Muhammed Mustahsen Rahman, Riham Mohamed, Sivan Padma Priya, Nallan CSK Chaitanya, Md Sofiqul Islam, Bakri Gobara

**Affiliations:** 1 RAK-College of Dental Sciences, RAK Medical & Health Sciences University, Ras Al Khaimah P.O. Box 12973, United Arab Emirates; mustahsin@rakmhsu.ac.ae (M.M.R.); riham.abdelraouf@rakmhsu.ac.ae (R.M.); sivan.padma@rakmhsu.ac.ae (S.P.P.); krishna.chytanya@rakmhsu.ac.ae (N.C.C.); sofiqul.islam@rakmhsu.ac.ae (M.S.I.); 2 RAK-College of Medical Sciences, RAK Medical & Health Sciences University, Ras Al Khaimah P.O. Box 11172, United Arab Emirates; rashababiker@rakmhsu.ac.ae; 3 Faculty of Dentistry, University of Khartoum, Khartoum 11115, Sudan; bakrigobara10@gmail.com

**Keywords:** periodontal diseases, gingivitis, periodontitis, microbial plaque biofilm, natural bioactive compounds, polyphenols, terpenoids, alkaloids, saponins

## Abstract

Periodontal diseases, chronic inflammatory conditions affecting oral health, are primarily driven by microbial plaque biofilm and the body’s inflammatory response, leading to tissue damage and potential tooth loss. These diseases have significant physical, psychological, social, and economic impacts, necessitating effective management strategies that include early diagnosis, comprehensive treatment, and innovative therapeutic approaches. Recent advancements in biomanufacturing have facilitated the development of natural bioactive compounds, such as polyphenols, terpenoids, alkaloids, saponins, and peptides, which exhibit antimicrobial, anti-inflammatory, and tissue regenerative properties. This review explores the biomanufacturing processes—microbial fermentation, plant cell cultures, and enzymatic synthesis—and their roles in producing these bioactive compounds for managing periodontal diseases. The integration of these natural compounds into periodontal therapy offers promising alternatives to traditional treatments, potentially overcoming issues like antibiotic resistance and the disruption of the natural microbiota, thereby improving patient outcomes.

## 1. Introduction

Periodontal diseases, including gingivitis and periodontitis, are major global health concerns, affecting over 50% of the population worldwide and accounting for substantial healthcare expenditures [1]. Epidemiologically, they are classified based on severity and extent, ranging from initial gingival inflammation to advanced periodontitis with severe tissue and bone loss [1,2]. Pathophysiological events in periodontal disease initiation involve microbial biofilm formation by pathogens like Porphyromonas gingivalis, leading to a host immune response characterized by the production of key pro-inflammatory cytokines, such as interleukin-1beta (IL-1β), produced by various immune and tissue-resident cells, including macrophages, oral fibroblasts, oral epithelial cells, and osteoblasts. The release of IL-1β induces the expression of other inflammatory cytokines and matrix metalloproteinases (MMPs), resulting in tissue breakdown and alveolar bone resorption [3,4]. The dysregulation of signaling pathways, including NF-κB and MAPK, leads to persistent inflammation and insufficient tissue regeneration [3]. The socioeconomic impact is significant, resulting in a lower quality of life, higher healthcare costs, and reduced productivity [5,6]. Effective management of periodontal disease requires a comprehensive understanding of these epidemiological and molecular aspects to develop targeted therapeutic strategies.

Periodontal therapy encompasses various modalities to prevent, manage, and treat periodontal diseases, including both non-surgical and surgical approaches and adjunctive treatments like antibiotics. However, antibiotics have drawbacks, such as antibiotic resistance and the disruption of the natural microbiota. Despite their efficacy, overreliance on antibiotics can lead to resistant bacterial strains, rendering them less effective over time. Additionally, broad-spectrum antibiotics can disrupt the natural balance of the oral microbiota, potentially leading to opportunistic infections or other oral health issues [7,8,9,10].

**Figure 1:**
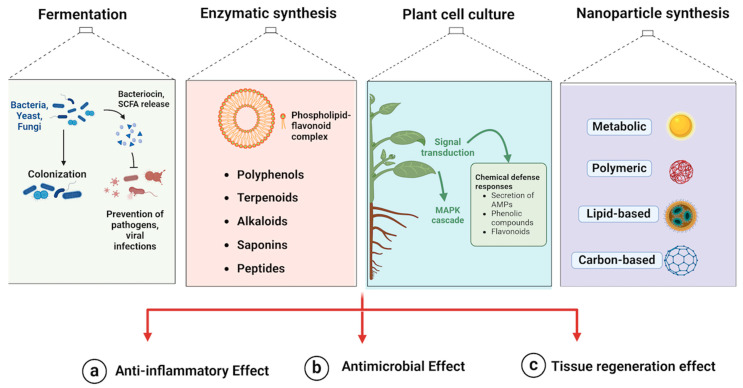
Illustrates the biomanufacturing processes for producing natural bioactive compounds for periodontal therapy, including fermentation, enzymatic synthesis, plant cell culture, and nanoparticle synthesis. Fermentation uses microbes to produce bacteriocins and SCFAs, preventing pathogen colonization. Enzymatic synthesis creates phospholipid–flavonoid complexes and various compounds. Plant cell culture utilizes signal transduction pathways to produce specific bioactive compounds. Nanoparticle synthesis involves creating diverse nanoparticles to enhance therapeutic delivery. These methods produce compounds with anti-inflammatory, antimicrobial, and tissue regeneration effects, demonstrating their role in improving periodontal health through advanced techniques. Created by Biorender.com.

Therapy involving biomanufacturing compounds offers a promising alternative. These compounds are derived from natural sources or produced through biotechnological processes, targeting specific pathogens associated with periodontal diseases while minimizing the risk of antibiotic resistance and preserving the balance of the oral microbiota. Furthermore, manufactured compounds can be engineered for enhanced efficacy and specificity, offering tailored treatment options for individual patients [11]. Integrating innovative approaches, such as biomanufacturing bioactive compounds, holds promise for enhancing periodontal therapy effectiveness and reducing the burden of these diseases [12]. Recent advancements in biomanufacturing have opened new avenues for producing natural bioactive compounds with potential therapeutic effects on periodontal diseases [13]. Biomanufactured compounds, derived from microbial fermentation, plant cell cultures, and enzymatic synthesis, have shown significant potential in managing periodontal diseases. These compounds include polyphenols, terpenoids, alkaloids, saponins, peptides, and nanoparticles, each exhibiting antimicrobial, anti-inflammatory, and tissue regenerative properties [14,15] (Figure 1).

This review aims to explore the biomanufacturing processes and the specific roles these bioactive compounds play in managing periodontal diseases, highlighting their potential as effective and sustainable therapeutic options.

## 2. Biomanufacturing Processes

### 2.1. Fermentation

Fermentation represents a powerful biomanufacturing process extensively utilized to produce bioactive compounds with various health benefits. Microorganisms such as bacteria, yeast, and fungi are employed to produce secondary metabolites with antimicrobial, anti-inflammatory, and antioxidant properties. In the context of periodontal diseases, fermentation holds significant promise in inhibiting periodontal pathogens and promoting oral health. For example, the production of bacteriocins by lactic acid bacteria through fermentation has shown promising results in inhibiting periodontal pathogens [16]. Fermentation involves the metabolic process of converting sugars into acids, gases, or alcohol using microorganisms such as bacteria, yeast, or fungi [17]. In the case of inhibiting periodontal pathogens, fermentation can be targeted toward specific microorganisms known to contribute to periodontal diseases [18]. Periodontal pathogens, such as Porphyromonas gingivalis, Tannerella forsythia, and Treponema denticola, thrive in the oral environment and contribute to the progression of periodontal diseases by causing inflammation and tissue destruction [19]. Fermentation processes can be optimized to produce bioactive compounds that target and inhibit the growth of these periodontal pathogens, thereby mitigating the progression of periodontal diseases [20].

Fermented bioactive compounds produced through fermentation processes can be utilized in various ways to combat periodontal diseases. Bacteriocins, which are antimicrobial peptides produced by bacteria through fermentation, exhibit selective antimicrobial activity against closely related species or strains. Bacteriocins produced by lactic acid bacteria have shown promise in inhibiting periodontal pathogens, such as Porphyromonas gingivalis and Prevotella intermedia. They possess a wide range of structural and functional variety, making them effective weapons against bacteria [21]. Derived from natural sources, bacteriocins exhibit stability even when exposed to high temperatures. Several recent studies have isolated and characterized bacteriocins for use in food technology. This application aims to prolong the shelf life of food, combat pathogenic diseases, aid in cancer treatment, and promote human well-being. Thus, bacteriocins can serve as a viable alternative to antibiotics for combating drug-resistant bacteria in the future [22].

Fermented bioactive compounds can be incorporated into oral care products, such as mouthwashes, toothpaste, and gels for daily oral hygiene routines. These compounds can also be formulated into targeted treatments, such as subgingival irrigation solutions or local drug delivery systems to deliver them directly to the most needed periodontal pockets. Incorporating fermented bioactive compounds into periodontal therapy regimens can complement traditional scaling and root planing treatments, providing a holistic approach to managing periodontal diseases [23].

### 2.2. Enzymatic Synthesis

Enzymatic synthesis involves using specific enzymes to catalyze the production of bioactive compounds, offering high specificity and mild reaction conditions that preserve the bioactivity of the compounds. Enzymes such as lipases and glycosyltransferases have produced compounds like flavonoids and saponins, exhibiting anti-inflammatory and antimicrobial activities [24]. This method aids periodontal therapy, as the enzymes’ high specificity allows for the precise control of reaction pathways and product formation, crucial for maintaining therapeutic properties [25,26].

In periodontal therapy, enzymatically synthesized flavonoids and saponins retain their beneficial activities, valuable for combating periodontal pathogens and inflammation. Additionally, enzymatic synthesis allows for the customization and optimization of reaction parameters, such as enzyme and substrate concentrations, reaction time, and temperature, to maximize the effectiveness of bioactive compounds [27,28]. This method is generally considered safe and environmentally friendly, eliminating the need for harsh chemicals and solvents used in traditional chemical synthesis [29,30]. Furthermore, enzymatic synthesis can produce various bioactive compounds beyond flavonoids and saponins, including peptides, oligosaccharides, and lipids, expanding the repertoire of treatment options available for periodontal health [31].

### 2.3. Plant Cell Cultures

Traditional harvesting practices to obtain functional phytochemicals often cause over-exploitation and environmental degradation. In this sense, plant cell culture (PCC)--based techniques are more promising for the sustainable production of bioactive compounds, with therapeutic potential in periodontal management. PCC enables the production of key bioactive compounds in a more controlled environment than common agricultural practices. Several alkaloids, terpenoids, and phenolic compounds with anti-inflammatory and antimicrobial activity can be efficiently produced by PCC [32,33]. PCC also enables the production of bioactive compounds in higher quantities and faster than traditional agricultural methods [34], ensuring the safety and efficacy of the final product, and the purity, potency, and consistency to meet regulatory standards [35] for clinical use in periodontal management. PCC’s efficiency ensures a reliable and consistent supply of therapeutic compounds for periodontal management.

### 2.4. Nanoparticle Synthesis

Nanoparticle synthesis involves the production of nanoparticles with unique physicochemical properties that enhance their bioactivity. These nanoparticles can be synthesized using various methods, including chemical reduction, sol–gel processes, and biological synthesis. Metallic nanoparticles (e.g., silver, gold), polymeric nanoparticles (e.g., chitosan, PLGA), lipid-based nanoparticles, and carbon-based nanoparticles are among those used in periodontal therapy [36].

## 3. Mechanisms of Action of Bioactive Compounds

Bioactive compounds exhibit various mechanisms of action that make them effective in managing periodontal diseases. These compounds interact with the lipid bilayer of microbial cell membranes, increasing membrane permeability, causing the leakage of cellular contents and ultimately cell death [37]. Additionally, certain bioactive compounds inhibit enzymes crucial for microbial metabolism and virulence, such as glucosyltransferases essential for synthesizing glucans in dental plaque [38]. They can also disrupt quorum sensing, a bacterial communication system that coordinates gene expression based on cell density, thereby inhibiting biofilm formation and virulence factor production. For instance, furanones from red algae interfere with the quorum-sensing signals of *Aggregatibacter actinomycetemcomitans*, reducing its virulence [39].

**Figure 2:**
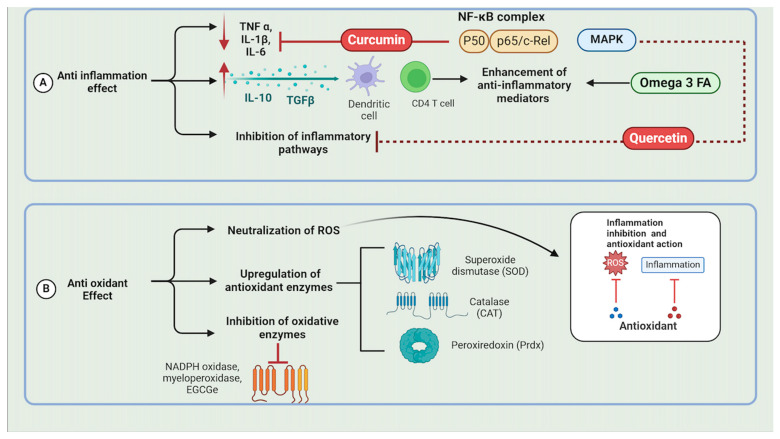
Illustrates the anti-inflammatory and antioxidant mechanisms of bioactive compounds in managing periodontal diseases. Curcumin inhibits the NF-κB complex, reducing pro-inflammatory cytokines, while quercetin and Omega-3 fatty acids reduce inflammatory mediators and enhance anti-inflammatory cytokines. Antioxidants neutralize reactive oxygen species, preventing cellular damage and enhancing endogenous antioxidant enzymes like SOD and CAT. Compounds such as EGCG from green tea inhibit oxidative enzymes, reducing ROS and oxidative stress, thus mitigating periodontal disease progression and promoting health. Created by Biorender.com.

Bioactive compounds also possess significant anti-inflammatory effects. They can inhibit the expression of pro-inflammatory cytokines such as TNF-α, IL-1β, and IL-6 by modulating signaling pathways like NF-κB and MAPK. Curcumin, a polyphenol derived from turmeric, exhibits its anti-inflammatory effects primarily through the inhibition of the NF-κB signaling pathway. NF-κB is a transcription factor that plays a critical role in regulating the immune response to infection. Curcumin inhibits NF-κB activation by preventing the phosphorylation and degradation of IκB, an inhibitor of NF-κB. This inhibition leads to a decrease in the production of pro-inflammatory cytokines, such as TNF-α, IL-1β, and IL-6, thereby reducing inflammation in periodontal tissues [40] (Figure 2).

Quercetin, a flavonoid found in many fruits and vegetables, exerts its anti-inflammatory effects through the inhibition of the MAPK signaling pathway. MAPKs are a family of serine/threonine protein kinases involved in various cellular activities, including proliferation, differentiation, and inflammation. Quercetin inhibits the MAPK pathway by preventing the phosphorylation of kinases, such as MEK1/2 and ERK1/2. This inhibition results in a reduced production of inflammatory mediators, thus attenuating the inflammatory response in periodontal tissues [41] (Figure 2). Furthermore, some bioactive compounds enhance the production of anti-inflammatory cytokines and mediators, such as IL-10 and TGF-β, promoting the resolution of inflammation. For instance, omega-3 fatty acids from fish oil increase the levels of IL-10, an anti-inflammatory cytokine, in periodontal tissues [42].

Additionally, bioactive compounds have antioxidant properties. They neutralize reactive oxygen species (ROS) by donating electrons, thus preventing oxidative damage to cellular components like lipids, proteins, and DNA [43]. They can enhance the expression and activity of endogenous antioxidant enzymes, such as superoxide dismutase (SOD), catalase, and glutathione peroxidase. Resveratrol, for example, upregulates the expression of SOD and catalase, enhancing the antioxidant defense in periodontal tissues [44] (Figure 2). Certain compounds also inhibit enzymes that produce ROS, such as NADPH oxidase and myeloperoxidase, thereby reducing oxidative stress. Epigallocatechin-3-gallate (EGCG) from green tea inhibits NADPH oxidase, reducing ROS production in periodontal tissues [45]. These diverse mechanisms underscore the potential of bioactive compounds in managing periodontal diseases through antimicrobial, anti-inflammatory, and antioxidant actions.

### Molecular Mechanism of Bioactive Compounds in the Management of Periodontal Diseases

- Polyphenols

Polyphenols, including flavonoids and phenolic acids, are known for their antioxidant and anti-inflammatory properties. Green tea catechins, such as epigallocatechin-3-gallate (EGCG), have been extensively studied for their ability to inhibit periodontal pathogens and reduce inflammatory mediators [46]. EGCG disrupts quorum sensing in *Aggregatibacter actinomycetemcomitans*, reducing its virulence and biofilm formation capability [47]. Additionally, EGCG inhibits NF-κB activation by preventing the phosphorylation and degradation of IκB, an inhibitor of NF-κB, leading to a reduced production of pro-inflammatory cytokines in periodontal tissues [48]. These actions help reduce the microbial load, modulate the host inflammatory response, and protect periodontal tissues from oxidative damage, thereby contributing to the overall management of periodontal diseases [49,50]. Polyphenols inhibit the phosphorylation and degradation of IκB, preventing NF-κB activation and subsequent pro-inflammatory cytokine production [51]. Polyphenols activate the Nrf2 pathway, leading to the increased expression of antioxidant enzymes and reduction of oxidative stress [52].

Polyphenols also have tissue regenerative properties, as they stimulate collagen synthesis by activating fibroblasts and increasing the expression of collagen genes [53]. For example, EGCG can specifically bind to receptors on fibroblasts, triggering intracellular signaling pathways that lead to cell activation and proliferation [46]. It also activates transcription factors, such as Smad and TGF-β (transforming growth factor-beta), which bind to the promoter regions of collagen genes (e.g., COL1A1, COL1A2). This binding enhances the transcription of these genes, leading to increased collagen production [46].

**Figure 3:**
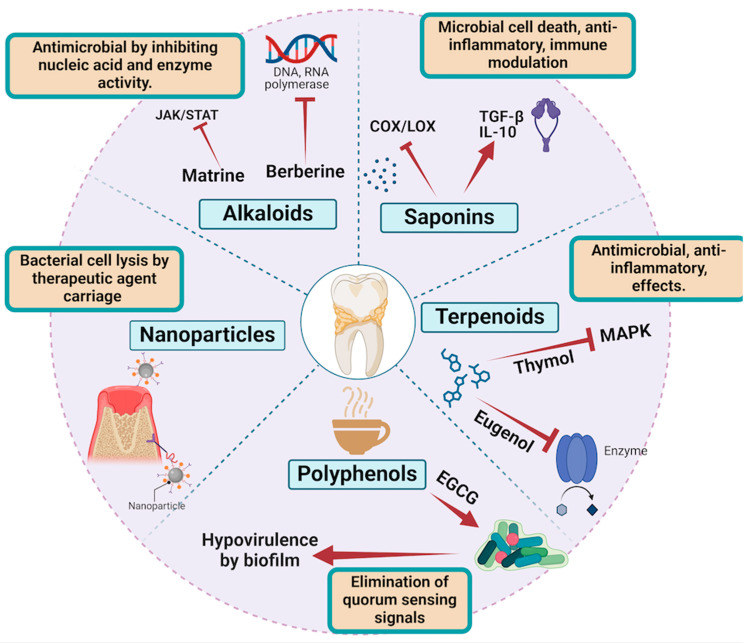
Describes the diverse bioactive compounds and nanoparticles used in periodontal therapy, highlighting their antimicrobial, anti-inflammatory, and biofilm-inhibiting effects. Alkaloids like matrine and berberine disrupt microbial nucleic acids and enzymes, while saponins compromise membrane integrity and inhibit inflammatory enzymes. Terpenoids such as thymol and eugenol provide dual antimicrobial and anti-inflammatory benefits. Polyphenols like EGCG from green tea reduce bacterial virulence and biofilm formation. Nanoparticles, including silver and chitosan-based nanoparticles, target bacterial cell lysis and biofilm disruption, collectively enhancing periodontal health by targeting pathogens and inflammatory pathways. Created by Biorender.com.

Polyphenols play a crucial role in periodontal therapy by promoting collagen synthesis through the activation of fibroblasts, the upregulation of collagen gene expression, the inhibition of MMPs, and antioxidant effects. These mechanisms contribute to the repair, regeneration, and maintenance of periodontal tissues, making polyphenols valuable therapeutic agents in managing periodontal diseases [14] (Figure 3).

- Terpenoids

Terpenoids, such as essential oils and their components (e.g., eugenol, thymol), exhibit antimicrobial and anti-inflammatory activities. These compounds can disrupt microbial biofilms and modulate the host immune response, making them valuable in periodontal therapy [54]. Eugenol and thymol integrate into the lipid bilayer due to their hydrophobic nature. This integration disrupts the membrane structure, increasing permeability, and causing the leakage of cellular contents [55]. The phenolic hydroxyl group of eugenol can form hydrogen bonds with lipid headgroups, further destabilizing the membrane [56]. Additionally, eugenol can bind to the active sites of enzymes such as ATPase and proteases, inhibiting their activity through hydrophobic interactions and hydrogen bonding with amino acid residues at the active site, leading to the loss of enzyme function and subsequent microbial growth inhibition [57].

Thymol inhibits the MAPK signaling pathway, which is involved in the production of inflammatory mediators. Thymol can bind to upstream kinases in the MAPK pathway, such as MEK1/2 and ERK1/2, preventing their phosphorylation and subsequent activation through hydrophobic interactions and hydrogen bonding with key residues in the kinase active sites [58]. Terpenoids, such as eugenol and thymol, exert their therapeutic effects in periodontal therapy through precise molecular interactions [59]. Their ability to disrupt microbial cell membranes, inhibit key enzymes, and interfere with quorum-sensing mechanisms provides potent antimicrobial activity. Additionally, their role in downregulating pro-inflammatory cytokines, inhibiting inflammatory pathways, and enhancing anti-inflammatory mediators contributes to their anti-inflammatory effects. These molecular interactions highlight terpenoids’ potential in effectively managing periodontal diseases [60] (Figure 3).

- Alkaloids

Alkaloids, including berberine and matrine, possess significant antimicrobial and anti-inflammatory properties [61]. Berberine, for example, has shown efficacy in inhibiting periodontal pathogens and reducing inflammation by modulating pro-inflammatory cytokines. Berberine intercalates into DNA, disrupting the replication and transcription processes [62]. This involves stacking interactions between the aromatic rings of berberine and the base pairs of DNA, stabilizing the DNA–berberine complex and inhibiting the function of DNA polymerase and RNA polymerase. Berberine inhibits the NF-κB pathway by preventing the phosphorylation and degradation of IκBα, an inhibitor of NF-κB. Berberine binds to IKK (IκB kinase), blocking its activity, preventing NF-κB from translocating to the nucleus, and promoting the transcription of pro-inflammatory cytokines like TNF-α, IL-1β, and IL-6 [63,64]. Matrine inhibits the JAK/STAT signaling pathway, reducing the production of cytokines involved in inflammation. Furthermore, Berberine enhances the expression of IL-10 by activating transcription factors like STAT3. This activation consists of the interaction of berberine with cell surface receptors that trigger STAT3 phosphorylation and nuclear translocation. Matrine increases the expression of TGF-β, an anti-inflammatory cytokine, through the Smad signaling pathway, promoting the resolution of inflammation [65,66]. Berberine and Matrine exert their antimicrobial and anti-inflammatory effects through specific molecular interactions, including membrane disruption, the inhibition of nucleic acid and enzyme activity, and the modulation of key inflammatory signaling pathways. These interactions make alkaloids valuable in the management of periodontal diseases by targeting both microbial pathogens and host inflammatory responses [62].

- Saponins

Saponins, found in various plants, exhibit antimicrobial, anti-inflammatory, and immune-modulating effects, making them valuable in managing periodontal diseases. Their amphiphilic structures, composed of hydrophilic glycoside moieties and hydrophobic aglycone (sapogenin) backbones, allow them to insert into microbial cell membranes. By forming complexes with membrane sterols like ergosterol in fungi, saponins disrupt membrane integrity, leading to increased permeability, the leakage of intracellular contents, and cell death [67,68]. Saponins also interfere with bacterial adherence to surfaces and the extracellular matrix production necessary for biofilm formation. They can disrupt established biofilms by penetrating the biofilm matrix and causing microbial cell death within the structure [69].

In addition to their antimicrobial properties, saponins inhibit enzymes such as cyclooxygenase (COX) and lipoxygenase (LOX), which are involved in the synthesis of pro-inflammatory mediators like prostaglandins and leukotrienes. This inhibition occurs through the direct binding of saponins to the active sites of these enzymes, blocking substrate access and catalytic activity [70]. Saponins also enhance the expression of anti-inflammatory cytokines, such as IL-10 and TGF-β, by activating transcription factors like STAT3 and Smad, mediated through receptor-ligand interactions that trigger signaling cascades, leading to the transcription of anti-inflammatory genes. For example, saponins from *Astragalus membranaceus* enhance IL-10 production by activating STAT3 in immune cells, promoting an anti-inflammatory environment. Moreover, saponins stimulate the activation and proliferation of immune cells such as macrophages, dendritic cells, and lymphocytes. This stimulation involves binding to cell surface receptors like Toll-like receptors (TLRs) and activating downstream signaling pathways that lead to immune cell activation and cytokine production [71]. For instance, Quillaja saponins activate dendritic cells through TLR2 and TLR4 signaling, enhancing their antigen-presenting capability and promoting adaptive immune responses. Saponins also increase the expression of phagocytic receptors and promote the production of reactive oxygen species (ROS) and nitric oxide (NO) in phagocytes. This enhancement involves activating signaling pathways like PI3K/Akt and MAPK, leading to increased phagocytosis and microbial killing [71]. For example, ginsenosides enhance the phagocytic activity of macrophages by upregulating the expression of scavenger receptors and promoting ROS production, leading to the efficient clearance of periodontal pathogens [72].

In summary, saponins exert their antimicrobial, anti-inflammatory, and immune-modulating effects through specific molecular interactions. Their amphiphilic structures enable them to disrupt microbial membranes and inhibit biofilm formation. By modulating key inflammatory signaling pathways and enhancing the production of anti-inflammatory cytokines, saponins reduce inflammation. Additionally, they activate and enhance the function of immune cells, improving the host’s defense mechanisms against periodontal pathogens. These molecular actions make saponins valuable agents in the management of periodontal diseases [73] (Figure 3).

- Nanoparticles

Nanoparticles have shown significant potential in managing periodontal diseases due to their unique properties, such as high surface area, tunable size, and the ability to carry and release therapeutic agents. Metallic nanoparticles, particularly silver nanoparticles (AgNPs), interact with the lipid bilayer of microbial cell membranes. AgNPs integrate into the lipid bilayer, causing increased membrane permeability and leading to the leakage of cellular contents. The silver ions released from AgNPs can interact with thiol groups in proteins and enzymes, further disrupting microbial functions [74]. Silver nanoparticles disrupt the cell membrane of *Porphyromonas gingivalis*, causing cell lysis and death [75] (Figure 3).

Nanoparticles offer a multifaceted approach to managing periodontal diseases through their antimicrobial, anti-inflammatory, and tissue regenerative properties. Chitosan and polymeric nanoparticles interfere with the extracellular polymeric substance (EPS) matrix production, inhibiting initial adhesion and biofilm maturation. Specifically, chitosan nanoparticles disrupt biofilm formation by *Aggregatibacter actinomycetemcomitans*, reducing bacterial colonization [76,77]. Additionally, nanoparticles such as cerium oxide (CeO_2_) and gold (AuNPs) can inhibit key signaling pathways involved in inflammation, including the activation of NF-κB and MAPK pathways. These nanoparticles can scavenge reactive oxygen species (ROS) and reduce oxidative stress, thereby decreasing the activation of these pro-inflammatory pathways [78]. CeO_2_ nanoparticles further inhibit NF-κB activation, reducing the production of pro-inflammatory cytokines like TNF-α, IL-1β, and IL-6 in periodontal tissues. With antioxidant properties, nanoparticles neutralize ROS; for instance, CeO_2_ nanoparticles exhibit superoxide dismutase (SOD) and catalase-mimetic activities, directly scavenging superoxide radicals and hydrogen peroxide [79,80]. This reduces oxidative stress in gingival fibroblasts, protecting them from ROS-induced damage [81].

Nanoparticles also promote the proliferation and differentiation of periodontal ligament cells, osteoblasts, and fibroblasts. Hydroxyapatite (HA) and bioactive glass nanoparticles release ions such as calcium and phosphate that enhance osteoblast activity and collagen synthesis, while providing a scaffold that supports cell attachment and growth [82]. These nanoparticles enhance osteoblast differentiation and mineralization, promoting bone regeneration in periodontal defects. Furthermore, polymeric nanoparticles such as PLGA can encapsulate antibiotics, anti-inflammatory drugs, and growth factors, protecting them from degradation and ensuring sustained release at the target site [83,84]. For example, PLGA nanoparticles loaded with doxycycline provide sustained antimicrobial activity and reduce inflammation in periodontal pockets [85]. By disrupting microbial membranes, generating ROS, inhibiting biofilm formation, modulating inflammatory pathways, scavenging ROS, promoting cell proliferation and differentiation, and enhancing drug delivery, nanoparticles provide a comprehensive strategy for periodontal therapy [86].

## 4. Clinical Applications of Bioactive Compounds

### 4.1. Topical Applications

Bioactive compounds can be formulated into mouthwashes, gels, and dentifrices for direct application to periodontal tissues. These formulations can provide localized effects, reducing microbial load and inflammation in the periodontal pocket [87].

### 4.2. Systemic Administration

Certain bioactive compounds can be administered systemically to exert their effects throughout the body. This approach can be beneficial in managing the systemic inflammation associated with periodontal diseases and improving overall periodontal health [88].

## 5. The Role of Bioactive Compounds and Nanoparticles in Periodontal Therapy

Recent research underscores the promising therapeutic potential of various bioactive compounds and nanoparticles for periodontal treatment. Studies have shown that curcumin and chitosan-based nanoparticles significantly reduce inflammation and improve periodontal healing [89,90]. Additionally, zinc oxide (ZnO) exhibits strong antibacterial properties without cytotoxic effects on fibroblasts, making it effective against periodontal pathogens [91]. Moreover, silver nanoparticles, particularly when used with scaling and root planing, show great promise in treating periodontal diseases [92]. Green tea extract is a beneficial adjunct in managing chronic periodontitis in both diabetic and non-diabetic individuals [93]. Furthermore, RES@PPD nanoparticles have demonstrated significant anti-inflammatory effects, enhancing their therapeutic potential for local inflammation [94]. Nanocrystalline hydroxyapatite (NCHA) emerges as an effective bone substitute material for periodontal regeneration, with outcomes comparable to conventional graft materials, such as bovine xenografts and synthetic alloplastic materials [95].

These findings collectively highlight the potential of incorporating these bioactive compounds and nanoparticles into standard periodontal therapies (Table 1). The consistency and robustness of these studies support their integration into clinical practice, thus paving the way for enhanced treatment outcomes and innovative solutions for managing periodontal diseases. Consequently, further research is encouraged to explore and validate these promising therapeutic strategies, ensuring their efficacy and safety in broader clinical applications.

## 6. Conclusions and Future Directions

The biomanufacturing of natural bioactive compounds presents a promising approach to managing periodontal diseases. Advanced production methods and a deeper understanding of the mechanisms of action enable these compounds to offer effective and sustainable therapeutic options. This review highlights the potential of manufactured natural bioactive compounds in periodontal therapy, emphasizing the importance of continued research and development in this promising field. However, there are several challenges and future directions that need to be addressed to fully realize their potential.

**Standardization and Quality Control**: One of the main challenges in the biomanufacturing of natural bioactive compounds is ensuring consistency in quality and efficacy. The standardization of production processes and stringent quality control measures are essential to produce reliable and effective therapeutic agents.

Clinical Trials and Regulatory Approval: Comprehensive clinical trials are necessary to evaluate the safety and efficacy of bioactive compounds in periodontal therapy. Navigating the regulatory approval processes is crucial to bring these novel treatments to market, ensuring they meet the necessary standards for clinical use.

Integration with Conventional Therapies: Future research should focus on integrating bioactive compounds with conventional periodontal therapies to enhance their efficacy. Combination therapies may provide synergistic effects, improving treatment outcomes for patients with periodontal diseases. This integration could lead to more comprehensive and effective treatment protocols, ultimately improving periodontal health and overall well-being.

By addressing these challenges and focusing on the future directions outlined, the potential of bioactive compounds in periodontal therapy can be fully realized, leading to innovative treatments that significantly improve patient outcomes.

## Figures and Tables

**Table 1:** Summary of studies using bioactive compounds and nanoparticles in periodontitis management. This table provides an overview of various studies conducted on the use of bioactive compounds and nanoparticles in the management of periodontitis, including details on the study titles, authors, publication years, types of compounds/nanoparticles used, and main findings.

Authors	Year	Bioactive Compound/Nanoparticle	Main Findings/Outcomes	Reference
Pérez-Pacheco, C.G., et al.	2021	Curcumin Nanoparticles	Reduced inflammation and improved periodontal healing compared to control.	[89]
Abhishek, K. Sah, et al.	2019	Chitosan-Based Nanoparticles	Chitosan-based drug delivery systems represent an attractive strategy for achieving the therapeutic concentration of drugs in periodontal pocket.	[90]
Griauzdyte, V.; Jagelaviciene, E.	2023	ZnO	The analysis of the literature confirms the antibacterial action of zinc against periodontal pathogenic bacteria. At low concentrations, these substances do not exhibit cytotoxic effects on fibroblasts.	[91]
Pooja K., et al.	2020	Silver nanoparticles	Silver nanoparticles gel with scaling and root planing gives promising results, and it can definitely aid in periodontal diseases	[92]
Gadagi, J.S., et al.	2013	Green tea extract	Local drug delivery using green tea extract could be used as an adjunct in the treatment of chronic periodontitis in diabetic and non-diabetic individuals.	[93]
Huangfu, H., et al.	2023	RES@PPD nanoparticles	RES@PPD NPs can remarkably decrease the level of pro-inflammatory cytokines, upregulate the anti-inflammatory cytokines, and exhibit a profound therapeutic effect on local inflammation.	[94]
Shaheen, M.Y.	2022	Nanocrystalline hydroxyapatite (NCHA)	NCHA is a suitable bone substitute material for periodontal bone regeneration, with outcomes comparable to that of conventionally used graft materials, such as bovine xenograft and other synthetic alloplastic materials.	[95]

## Data Availability

All data are contained within the article.
